# Piloting an automated clinical trial eligibility surveillance and provider alert system based on artificial intelligence and standard data models

**DOI:** 10.1186/s12874-023-01916-6

**Published:** 2023-04-11

**Authors:** Stéphane M. Meystre, Paul M. Heider, Andrew Cates, Grace Bastian, Tara Pittman, Stephanie Gentilin, Teresa J. Kelechi

**Affiliations:** 1 OnePlanet Research Center and imec, Toernooiveld 300, Nijmegen, 6525 EC The Netherlands; 2 Medical University of South Carolina, Charleston, SC USA

**Keywords:** Medical informatics [L01.313.500], Natural language processing (NLP) [L01.224.050.375.580], Data science [L01.305], Clinical trial enrollment/recruitment

## Abstract

**Background:**

To advance new therapies into clinical care, clinical trials must recruit enough participants. Yet, many trials fail to do so, leading to delays, early trial termination, and wasted resources. Under-enrolling trials make it impossible to draw conclusions about the efficacy of new therapies. An oft-cited reason for insufficient enrollment is lack of study team and provider awareness about patient eligibility. Automating clinical trial eligibility surveillance and study team and provider notification could offer a solution.

**Methods:**

To address this need for an automated solution, we conducted an observational pilot study of our TAES (TriAl Eligibility Surveillance) system. We tested the hypothesis that an automated system based on natural language processing and machine learning algorithms could detect patients eligible for specific clinical trials by linking the information extracted from trial descriptions to the corresponding clinical information in the electronic health record (EHR). To evaluate the TAES information extraction and matching prototype (i.e., TAES prototype), we selected five open cardiovascular and cancer trials at the Medical University of South Carolina and created a new reference standard of 21,974 clinical text notes from a random selection of 400 patients (including at least 100 enrolled in the selected trials), with a small subset of 20 notes annotated in detail. We also developed a simple web interface for a new database that stores all trial eligibility criteria, corresponding clinical information, and trial-patient match characteristics using the Observational Medical Outcomes Partnership (OMOP) common data model. Finally, we investigated options for integrating an automated clinical trial eligibility system into the EHR and for notifying health care providers promptly of potential patient eligibility without interrupting their clinical workflow.

**Results:**

Although the rapidly implemented TAES prototype achieved only moderate accuracy (recall up to 0.778; precision up to 1.000), it enabled us to assess options for integrating an automated system successfully into the clinical workflow at a healthcare system.

**Conclusions:**

Once optimized, the TAES system could exponentially enhance identification of patients potentially eligible for clinical trials, while simultaneously decreasing the burden on research teams of manual EHR review. Through timely notifications, it could also raise physician awareness of patient eligibility for clinical trials.

**Supplementary Information:**

The online version contains supplementary material available at 10.1186/s12874-023-01916-6.

## Background

Insufficient patient enrollment in clinical trials remains a serious and costly problem and is often considered the most critical barrier to their timely execution [1]. Trials that fail to meet patient recruitment goals can cause delays, lead to early trial termination, or make it impossible to draw conclusions at trial completion due to insufficient statistical power. In a study of accrual patterns in four U.S. cancer treatment centers, Dilts and Sandler observed that almost 60% of trials opened for five years had fewer than five patients enrolled at each site, and in more than 20% of studies, not a single participant had been accrued [2]. These low- or zero-enrolling trials waste investigators’ time, jeopardize research funding, and expose patients to risks inherent in each study without offering scientific insight.

Despite the urgent need for increased recruitment, most patients are never offered an opportunity to enroll in clinical trials. In a recent systematic review of 9,675 published studies, 13 of which met inclusion criteria, only an average of 8% of patients were enrolled, while an additional 70% were eligible but not offered a trial for various reasons [3]. Particularly low enrollment levels have been reported for oncology patients (< 5%) and patients diagnosed with COVID-19 (4%) [4–6]. Data show insufficient enrollment is biased towards women, African Americans and Native Americans, who are under-represented in new treatment trials, even those aimed at diseases that disproportionately affect them [7, 8].

This failure to approach patients about clinical trials is all the more unfortunate because most patients and providers are in favor of trial participation. Surveyed providers strongly agree (86.1%) or somewhat agree (9.7%) that clinical trials provide high-quality care and agree (88.7%) that trials benefit enrolled patients [9]. Other survey data show that 94% of patients have expressed willingness to participate in clinical trials, [10] especially when trials are recommended by a healthcare provider. Unfortunately, lack of provider awareness of their patients’ eligibility for clinical trials is an oft-cited reason for low enrollment [11].

Tapping into the data stored in electronic health records (EHRs) could help to address this problem. A recent survey of Clinical and Translational Science Awards (CTSA) consortium members confirmed interest in using EHR data to support trial recruitment [12]. Using the EHR or registries to identify eligible patients and then alerting providers or trial staff of their eligibility was cited as the most effective solution for raising awareness and increasing enrollment in another recent survey of trial stakeholders, including sponsors, investigators, study coordinators and patient advocacy groups [13]. Any such solution would need to integrate seamlessly into providers’ workflow and not add to their digital burden.

### Automating clinical trial eligibility screening

Screening patients manually is typically a cumbersome and lengthy process, with screening times ranging from about 10 min per trial per patient for criteria with minimal complexity to more than two hours for highly complex sets of criteria [14]. The time required for a chart review has only increased with the exponential growth in patient information made available by the advent of EHRs. A variety of automated approaches have been proposed to alleviate the burden of manual eligibility screening on research teams. Initially, most of the automated solutions were based on structured and coded information from the HER [15, 16], pager notifications, [17, 18] alerts and clinical decision-support system integration, [19] and advertising (e.g., using Facebook) [20]. These efforts relied on manual definitions of the eligibility criteria, resulting in an incomplete automated solution that could not be easily scaled. Another limitation of these systems was their reliance exclusively on structured data, when most of the information about clinical trial eligibility in protocols (e.g., Clinicaltrials.gov) and the corresponding patient clinical information in the EHR are found in unstructured narrative text. For example, in a recent experiment focused on breast cancer trials, 96% of information on eligibility criteria was mentioned only in narrative text [21]. To unlock the data in the narrative text of protocols and electronic patient records, some have proposed automated solutions using natural language processing (NLP), which can “read” unstructured, narrative text and transform it into structured data.

### A short history of NLP used for screening trial eligibility

Several experiments have applied NLP and other information extraction methods to collect eligibility criteria automatically from narrative text. These experiments focused either on trial protocols or on clinical notes. For the former, NLP was applied to extract generic query patterns representative of eligibility criteria [22]. Tian et al. compared several deep neural network models to extract mentions of 11–15 categories of eligibility criteria [23]. Weng et al. developed the EliXR system to extract eligibility criteria from ClinicalTrials.gov trial protocols [24] and then modified it to export the extracted criteria in the Observational Medical Outcomes Partnership (OMOP) common data model (CDM) format (EliIE system [25]) to make it accessible through a web application (Criteria2Query [26–28]). Beck and colleagues used IBM® Watson for Clinical Trial Matching (WCTM) to extract eligibility criteria from four breast cancer trial protocols and help to enroll patients after manual verification [29]. Helgeson et al. also used it for four breast and three lung cancer trials [30].

The first system to retrieve trial eligibility-relevant information automatically from clinical notes in the EHR used simple pattern matching to extract information from surgical pathology reports [31]. At the 2011 and 2012 Text Retrieval Conferences (TREC), teams of researchers competed to identify clinical notes matching simple eligibility criteria (e.g., “Elderly patients with subdural hematoma”) [32]. More recent efforts focused on children visiting the emergency department [33] or potentially eligible for a selection of cancer trials [34]. These efforts eventually resulted in the integration of the Automated Clinical Trial Eligibility Screener system into the clinical research coordinators’ workflow in the emergency department [35]. The National NLP Clinical Challenges (n2c2) provided another opportunity for teams of researchers to compete to automate clinical trial cohort selection by requiring them to identify sets of clinical notes matching any of 13 eligibility criteria [36].

### The trial eligibility surveillance system

We hypothesize that a more complete automated solution would provide end-to-end integration between the recognition of eligibility criteria in trial protocols and eligibility information found in patient EHR records using NLP and other applications of machine learning algorithms. It would also provide accurate and adaptable matching between the two sets of information by adopting a common data model for both.

In 2018–2019, the Medical University of South Carolina (MUSC) and Hollings Cancer Center (Charleston, SC) piloted a breast cancer trial enrollment support system developed by Meystre and colleagues known as The TriAL Eligibility Surveillance (TAES, pronounced “ties”) system [21, 37]. TAES uses NLP and other machine learning algorithms to extract patient information from EHR clinical notes and structured sources and compare it with eligibility criteria extracted from trial protocols. Here, we enhanced TAES to be more relevant to a broader range of clinical trials and piloted the TAES information extraction and matching prototype (i.e., TAES prototype) in five open cardiovascular and cancer trials to test whether an automated process based on NLP and machine learning algorithms could detect patients eligible for specific clinical trials by linking the information extracted from trial descriptions to the corresponding clinical information in the EHR. We also developed a simple web application to interact with a new database storing all trial eligibility criteria, corresponding clinical information and trial-patient match characteristics using the OMOP CDM. Finally, we investigated options for integrating TAES into the EHR and for automating prompt notification of health care providers of potential patient eligibility without interrupting their clinical workflow.

## Methods

Three objectives guided the design of the pilot study of the TAES prototype. First, we aimed to implement an automated trial eligibility surveillance system that would extract and normalize clinical information from structured and unstructured EHR content and match it with normalized eligibility criteria from clinical trial protocols. Second, we wanted to develop a user interface for researchers to access the trial-matching database, select clinical trials, review the extracted eligibility criteria, define patient populations, and examine matching patient records along with the available evidence used to determine possible eligibility automatically. Finally, we wanted to assess options for connecting the trial eligibility surveillance system with a commercial EHR system for provider (and patient) notification.

### Automated trial eligibility surveillance system

**Fig. 1:**
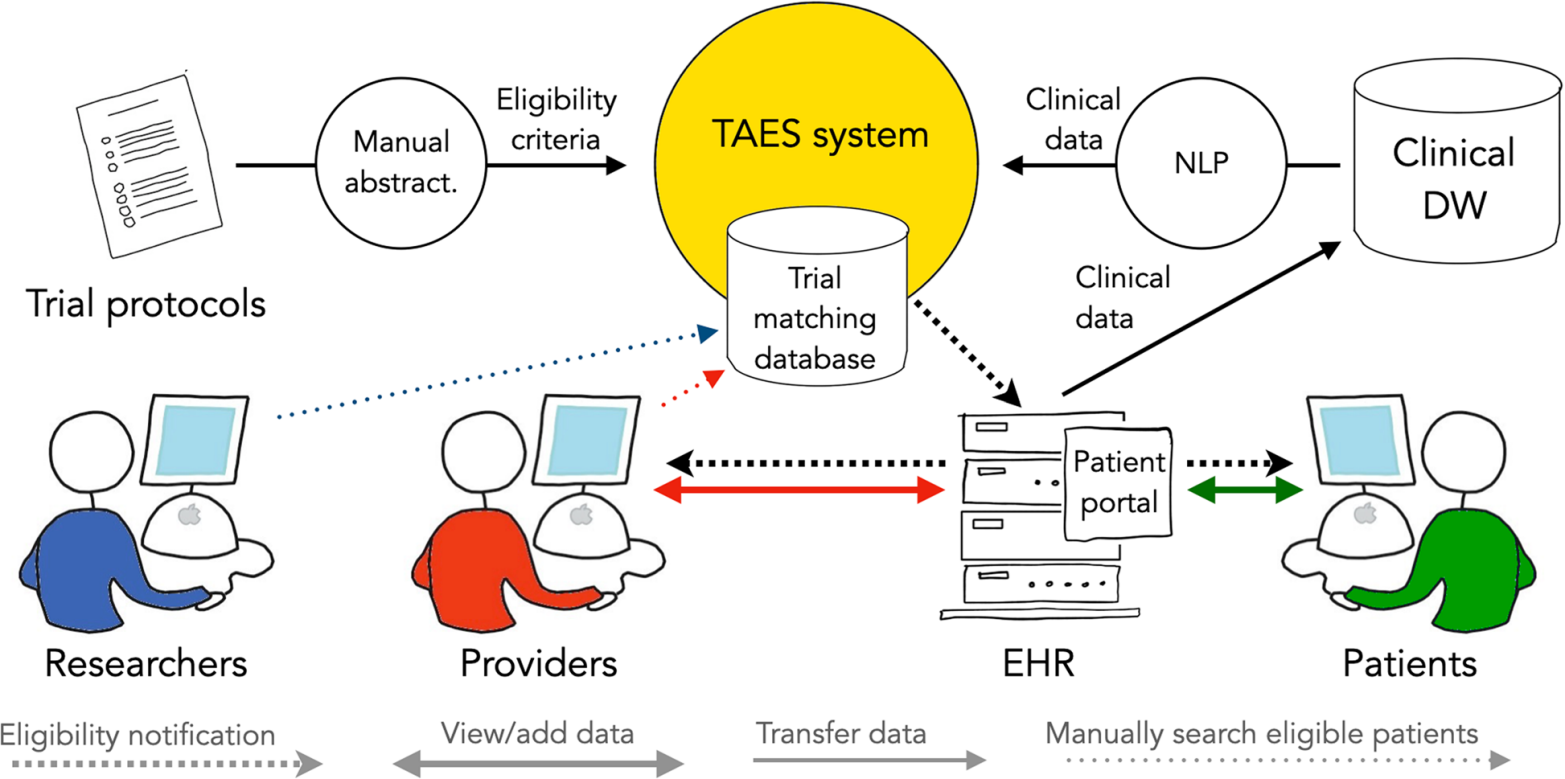
Trial Eligibility Surveillance (TAES) system overview. DW = data warehouse; NLP = natural language processing

As depicted in the top section of Fig. 1, the TAES prototype detects potentially eligible patients by acquiring trial eligibility criteria from protocols, extracting clinical information from the EHR, and identifying matches between the two sets of information.

For abstraction of trial eligibility criteria, we selected five cardiovascular and cancer trials open for enrollment at MUSC with at least ten enrolled participants. All eligibility criteria, regardless of their likelihood to be extracted by an NLP system (as discussed in "Study limitations" below), were then retrieved from study protocols (or other sources) and manually abstracted using an electronic tool that enables domain experts to represent eligibility criteria in a structured and coded form (ATLAS open-source software tool, available from the Observational Health Data Sciences and Informatics (OHDSI) consortium [38]. We use the OMOP CDM with a selection of standard terminologies for representing these criteria, as this is a well-established model.

Clinical information stored in MUSC’s EHR is currently exported in real time or daily to an institutional clinical data warehouse. The TAES prototype extracts clinical information corresponding to eligibility criteria from EHR notes and represents this information with the same CDM and terminologies as the eligibility criteria in the trial-matching database. Extracted concepts can be grouped into six categories: conditions or diseases, investigations, medications, procedures, devices, and demographics. The “conditions and medications” extraction was enriched with six binary contextual attributes, which indicated if the extracted information was negated, uncertain, conditional, generic, historical, or not about the patient. Since a majority of the clinical information is recorded in narrative text only, we also used NLP to extract structured and coded information. We used DECOVRI (Data Extraction for COVID-19-Related Information) as the NLP tool for information extraction, a locally-developed and freely available open-source NLP tool built on Apache UIMA [39, 40]. DECOVRI was originally developed to extract COVID-19 related information, but it’s modules for extracting medications, demographics, and contextual attributes were considered sufficient for this task (i.e., good accuracy was measured with similar information extracted from clinical text notes in previous evaluations of DECOVRI, with gender and age extracted with 100% recall and medication attributes extracted with 68–98% recall). To adapt it to this new task, we added custom lexicons for conditions, procedures, investigations, and devices. These lexicons were generated with lex_gen, a freely available open-source tool that uses the UMLS Metathesaurus relations to create rich lexicons from a seed set of concepts [41].

Eligibility information is stored in the trial-matching database, along with all supporting data, for subsequent access. In this pilot study, we used the rule-based approach we experimented with earlier to assess trial eligibility [21]. This approach uses rules implemented as database queries exported from ATLAS and then applied in a database management tool. We first evaluated the available structured coded information relevant to trial eligibility criteria as a baseline. We then evaluated how the inclusion of information extracted by our NLP system improved or honed the review process for finding likely eligible patients. The queries were used to determine how many individual eligibility criteria a patient met for a given trial out of all possible criteria. The maximum score (e.g., 12 if there were 12 criteria) means all criteria are met, while a score of zero means no criteria are met.

Domain experts, including medical residents and advanced medical students with clinical documentation experience, built a reference standard based on the five selected trials to measure the accuracy of the automated patient eligibility assessment. We used historical enrollment decisions for a stratified random selection of 400 patients (including at least 100 enrolled in the selected trials) in the reference standard. The domain experts reviewed the EHR records of the selected patients and annotated information matching eligibility criteria using a secure web-based annotation tool (INCEpTION [42]). To guide their annotations, experts were provided with an annotation schema that matched the six general extraction categories described above. Annotators were also asked to flag any medication indicated as an allergen and annotate non-medication allergen mentions. For the detailed evaluation of the information extraction process, a random selection of 20 text notes from the aforementioned dataset was annotated in detail (i.e., all corresponding text spans and local context information). We then compared this reference standard with the extracted clinical information and the automatic eligibility classification to measure sensitivity, positive predictive value, and the area under the ROC curve (AUC).

### Trial-matching database and web application

**Fig. 2:**
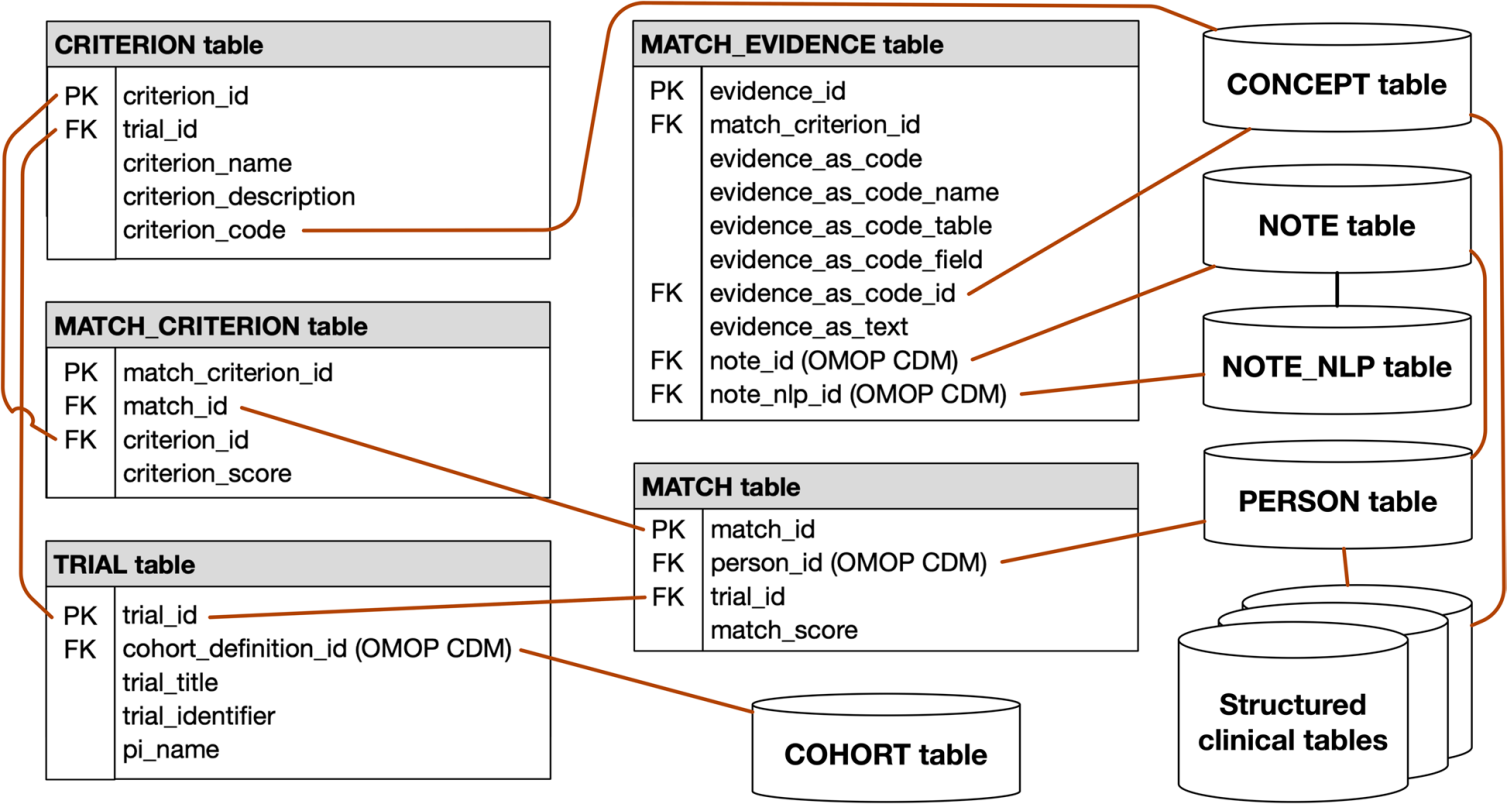
Trial Eligibility Surveillance (TAES) database schema

The trial-matching database includes eligibility criteria, potentially eligible patients, and the patient information that matched eligibility criteria. The database is mostly based on the OMOP CDM, with the addition of custom tables dedicated to trial-patient matching information. Figure. 2 provides an overview of the database architecture, with links to the pre-defined tables in the OMOP CDM. The TRIAL table contains metadata (e.g., the name of the study’s principal investigator) that does not fit into the OMOP CDM’s COHORT table. The CRITERION table includes executable definitions for each eligibility criterion defined in a trial. For this pilot study, we embedded the database query code to extract all patients matching a specific criterion as the executable definition. The query result provides the rows of evidence to be added to the MATCH_EVIDENCE table. All individual instances of evidence for a patient, criterion, and trial triplet are aggregated into a single summary score in the MATCH_CRITERION table. Likewise, all individual criterion scores for a patient and trial pair are aggregated into a single summary score in the MATCH table. These scores help filter matches so that study teams can focus on the most promising ones.

A user-friendly web application provides access to trial-patient matching information, clinical trial search and selection, potentially eligible patients for further screening, and a visualization of matching patient records along with the available evidence used to a determine possible eligibility automatically (e.g., diagnostic or treatment code or information highlighted in the text note from which it was extracted). The web application was developed using the flexible Ruby on Rails platform with a Bootstrap [43] front end to simplify the user experience while providing a robust, elegant platform on which to build. The web application was designed to enable users to search, identify, and flag potentially eligible patients quickly. The selected matches could then be easily exported for further eligibility screening.

The OHDSI WebAPI enables interactions with the OMOP CDM database of extracted patient and trial information [44]. The OHDSI ATLAS platform provides access to the OMOP CDM database for detailed data exploration and population analysis, terminology browsing, cohort definition, and other database queries.

### Exploration of options for connecting to commercial EHR systems

For this pilot study, members of the MUSC biomedical informatics and information systems teams considered a variety of options for integrating information from the TAES trial-matching database into the Epic EHR used at MUSC. They also considered options for communicating matches to healthcare providers (i.e., clinical workflow integration). Options were identified from electronic documentation and summarized into strategies and subsequent procedures. The potential strengths and weaknesses of each option were analyzed. Findings were then presented and discussed with an ad hoc trial eligibility notification stakeholders’ group that was created to guide integration efforts. The overarching aim was to explore possible options to integrate TAES with a commercial EHR system to then make providers aware of patient trial eligibility as early as possible during a clinical encounter, using documentation tools familiar to physicians so as not to disrupt workflow. In the future, patients interested in such notifications could also enroll through the EHR patient portal (e.g., MyChart for the Epic EHR system).

## Results

### Automated trial eligibility surveillance system

A variety of cardiovascular and oncology trials were selected for the pilot study (Table 1). The eligibility criteria for the selected studies were manually abstracted and included between 2 and 12 concepts (e.g., arrhythmogenic right ventricular cardiomyopathy [SNOMED CT concept 281170005]), with 7 to 29 rules (e.g., include children of the selected concept, age ≥ 18 years, exclude all instances of a specific concept, concept A must happen before concept B) for each study. They were all exported from the ATLAS tool in JSON format for use in the TAES system.

**Table 1:** Clinical trials included in the study

Trial title	ClinicalTrials.gov ID
FLExAbility Sensor Enabled Substrate Targeted Ablation for the Reduction of VT Study (LESS-VT)	NCT03490201
Dapagliflozin Evaluation to Improve the LIVEs of Patients With PReserved Ejection Fraction Heart Failure (DELIVER)	NCT03619213
Study of Chitosan for Pharmacologic Manipulation of AGE (Advanced Glycation End products) Levels in Prostate Cancer Patients	NCT03712371
Durvalumab With Radiotherapy for Adjuvant Treatment of Intermediate Risk SCCHN	NCT03529422
Efficacy of narrow band UVB phototherapy for cutaneous graft-versus-host disease in allogenic hematopoietic stem cell transplant recipients	N/A

The rapid development of the TAES prototype using DECOVRI required creation of four custom lexicons: diseases/conditions, investigations (other than laboratory test results), procedures, and devices. The “medication extraction and laboratory test result” components (listed with investigations) were reused from earlier research [39, 45] without adaptation.

The reference standard built from the stratified random selection of 400 patients includes 21,974 clinical notes of various type and size (average of 423 words, with a minimum of 1 word and a maximum of 9,490 words). The small subset of 20 notes annotated in detail includes 1,047 concept annotations with local context attributes (details in Table 2). Compared with this small subset of annotated notes, the performance of the TAES prototype was moderate, with a measured micro-averaged recall of 0.514 (0–0.624) and a micro-averaged precision of 0.624 (0–1.000; Table 2). Performance was mostly moderate and varied largely with recall between 0 and 0.778, and precision was between 0 and 1.000 (Table 2).

**Table 2:** Accuracy of information extraction

	**Annotations in the reference**	**True positive**	**False positive**	**False negative**	**Recall**	**Precision**	**F**_**1**_**-measure**
Disease/Condition	270	136	207	134	0.504	0.397	0.444
Investigation name	194	108	72	86	0.557	0.600	0.578
Investigation result value	167	70	6	97	0.419	0.921	0.576
Medication	200	191	36	99	0.659	0.841	0.739
Procedure	75	3	0	72	0.040	1.000	0.077
Age	22	16	0	6	0.727	1.000	0.842
Gender	18	14	3	4	0.778	0.824	0.800
Device	10	0	0	10	0.000	0.000	0.000
**Micro-average**					**0.514**	**0.624**	**0.564**
**Macro-average**					**0.409**	**0.798**	**0.579**

In this pilot study, we only evaluated the trial-patient match accuracy for one of the selected clinical trials: the narrowband type B ultraviolet (NBUVB) phototherapy study listed in Table 1. We had 6,297 clinical notes associated with 9 patients who were known to be eligible and an additional 159,627 notes associated with 301 patients with unknown eligibility. All of the 310 patients had a coded diagnosis of graft-vs-host disease (GVHD), and none had structured coded procedure codes for NBUVB therapy. We then wrote SQL queries against the OMOP CDM NOTE_NLP table in the trial-matching database (where extracted data was stored by the NLP system) to identify patients with affirmed and negated mentions of a GVHD diagnosis and NBUVB therapy exposure. The NLP system successfully extracted evidence from the notes for GVHD and NBUVB from all 9 of the 9 patients known to be eligible. Of the remaining 301 patients of unknown eligibility, the NLP system extracted evidence for a GVHD diagnosis in only 294 (97.7%) of the patients, indicating that even though all patients had coded diagnoses, the diagnosis may not always be indicated in the unstructured text note. Further, 30 of the patients (10.0%) had evidence for NBUVB therapy.

### Trial-matching database and web application

**Fig. 3:**
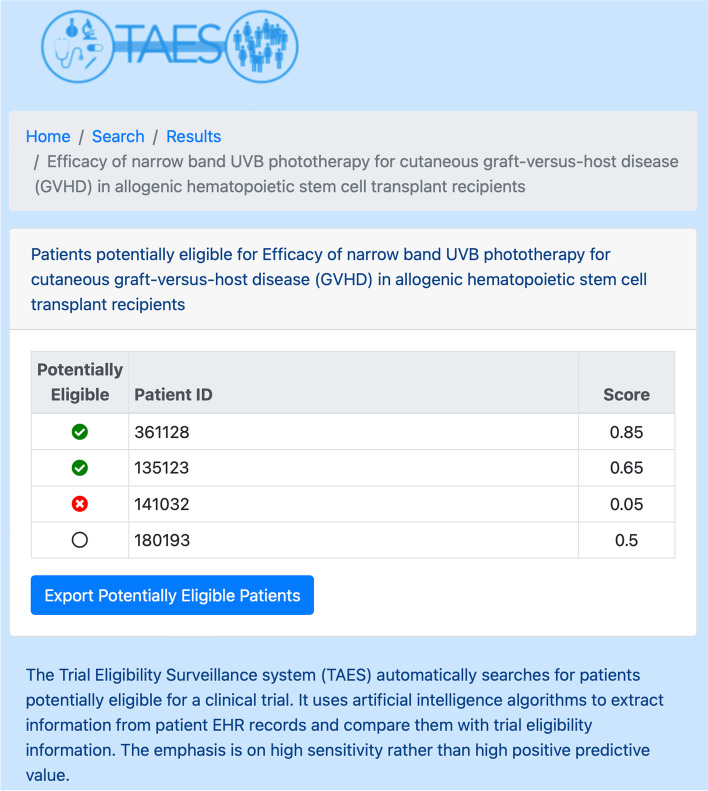
Web application for accessing the Trial Eligibility Surveillance (TAES) matching database

The simple web application developed to interact with the trial-patient matching database provides a number of important functionalities. It can search for a clinical trial using the trial identifier or the name of the trial or principal investigator, display details on the selected trial for verification, and list all patients matching the selected trial criteria using an anonymous identifier and an overall match score (Fig. 3). It also enables study teams to identify the match between eligibility criteria and patient information and provides them with de-identified evidence on the basis of which they can select or exclude patients listed as potentially eligible (Fig. 3). Finally, with proper authorization, it can export a list of potentially eligible patients for subsequent re-identification and recruitment. The TAES web application can be accessed using the MUSC single sign-on infrastructure (based on Shibboleth).

### Exploration of options for connecting to commercial EHR systems

We considered a variety of options for the technical and workflow integration of the TAES system. For technical integration, options included SmartData Elements, the creation of a study record in the EHR, Best Practice Advisory (BPA) web services [46], and a new integrated web application. SmartData Elements would require the use of private Epic tools, such as Clarity® Datalink or Private Epic application programming interfaces (APIs), to update the TAES system or create match records accessible in the Epic EHR. Creating a specific study record in the Epic EHR would allow linking with the Epic MyChart patient portal using open-source standards such as CDS-Hooks or SMART on FHIR. A call from the EHR system could be triggered automatically upon a defined workflow action (e.g., patient visit started, an order placed) or on-demand with a clickable button [47]. The integrated web application would offer the most opportunity for customization and could be accessed using a configured button or link in the Epic EHR. The TAES application interface would then launch in a designated frame or window. For clinical workflow integration, we considered BPAs triggered by actions (e.g., click, order), print groups, summary reports listing patients with select information (e.g., trial eligibility) to be shared at the beginning of a consult section, MyChart notification of patients, and the launch of a custom application (as above).

Stakeholders were invited to provide input on the location and display of information and links in the user interface. For example, they could state their preferences as to where matches would be displayed and in what format. Options for context-specific display locations included “in a patient’s chart,” “during an encounter,” and “for the logged-in provider.” Potential locations for a general button linking to the TAES application interface included top-level and sidebar menus. Alternatively, reports of matches could be run in the EHR system as needed, using built-in reporting tools. When these options were discussed with members of the trial eligibility notification stakeholders’ group, most agreed that providers would not like alerts or interruptions for trial eligibility. They thought that solutions that do not interrupt the ongoing workflow, such as "soft" BPAs (only listed on the user interface side), would be more appropriate. Principal investigators and study coordinators would be motivated to review and filter these alerts, forwarding them to relevant providers only (e.g., selected by specialty or role). Since the adoption of a default opt-in approach at MUSC in 2021, provider authorization has not been required before contacting patients about trial recruitment.

Any integration approach will require at least some configuration of the EHR system. Details on how to implement the options described above in Epic are available to organizations using the Epic EHR via the referenced documentation on the Epic UserWeb or through the Epic Technical Services representative.

## Discussion

We implemented a simple version of the TAES system using mainly existing resources. This pilot study of the TAES prototype showed that NLP can be used to collect relevant trial eligibility data locked in the narrative text of trial protocols (i.e., eligibility criteria) and the EHR (i.e., corresponding patient information) with moderate accuracy, offering an end-to-end solution. Both sets of data were then transformed into OMOP-CDM format for automated matching and stored in a matching database for researchers to access via a simple Web application. The OMOP-CDM was chosen for its growing popularity, especially among academic healthcare institutions, and for the availability of tools easing the capture, storage, querying, and analysis of data using this format. As prominent example of this popularity, the National COVID Cohort Collaborative (N3C [48]), uses the OMOP-CDM as its core data model, with mappings to other popular data models such as PCORnet.

The pilot study also successfully assessed options for integrating an automated clinical trial eligibility surveillance system into the EHR and the clinical workflow. It found that providers preferred “soft” BPA notifications (listed only on the user interface side) that allowed researchers to screen potential matches before contacting providers.

### Performance and errors analysis

The results of the pilot study point to the need for improvements in NLP-based information extraction. For “diseases/conditions,” both recall and precision were insufficient. A new lexicon will be needed to ensure more selective coverage of all concepts included in trial eligibility criteria, and this includes concepts with hierarchical relations in the UMLS Metathesaurus (e.g., C0018133 “Graft-vs-Host Disease” parent of C1610605 “Graft versus host disease in skin”). For “investigation names,” the existing lexicon will be expanded to include missing content. The “laboratory test names and results extraction” component was reused without adaptation, but several concepts were missed. Re-training of these deep neural network-based components will be needed. For “medications extraction,” precision was satisfying but recall was insufficient. Several medications were missed, and the “medication extraction” component that was reused without adaptation will also need to be retrained. For “procedures and devices,” a clear lack of coverage of the lexicon was observed (recall: 0.04 and 0.00, respectively).

While these lexicons correctly reflected the devices relevant to the specific trial eligibility criteria, the variety of other devices discussed in a patient’s note was not well covered. In other words, these initial generated lexicons were overly specific to the task at hand and would need to be generalized or broadened in future work.

When evaluating the trial-patient match accuracy, we used existing coded information as a reference resulting in 100% sensitivity for the GVHD diagnosis among enrolled patients, and 97.7% sensitivity among patients with unknown eligibility. These findings indicate that the diagnosis was not always mentioned in the unstructured text note, an expected outcome considering the variety of clinical notes we included. For NBUVB therapy information, we extracted this information from clinical notes of 10% of the patients without this coded procedure and interpret this performance as a benefit rather than a failure of TAES. Namely, without application of the NLP system, a manual chart review of all 301 patients’ notes would be required to find the eligible subset. With the application of the NLP system, we have a subset of 30 patients with a strong likelihood of being eligible and a second subset of 271 patients with a lesser likelihood.

### Study limitations

This pilot study has several limitations to consider. First, only a small subset of our large 21,974 clinical notes dataset was used for patient eligibility assessment and extraction of information from clinical text. That small sample size permitted an assessment of the capabilities of the TAES prototype and its integration but limited our ability to demonstrate the higher potential accuracy machine learning could offer and caused limited information extraction accuracy for rare information types. Second, only a subset of the eligibility criteria listed for each trial was selected for matching with EHR data. This partial selection was based on the criteria considered most important by the domain experts, on the availability of the data in the EHR, or the criteria allowing for high sensitivity rather than high precision when searching for potentially eligible patients.

## Conclusion

Once fine-tuned, our proposed NLP-based automated clinical trial eligibility surveillance system could exponentially enhance identification of patients potentially eligible for clinical trials. It could do so while reducing the burden of manual EHR review on study teams. It could also raise provider awareness of patient eligibility and increase the ease and efficiency of their involvement in patient notification and recruitment.

## Supplementary Information

**Additional file 1. **

## Data Availability

The datasets generated and/or analyzed during the current study are not publicly available due to their privacy protection requirements (patient personal healthcare information, even if de-identified) but are available from the corresponding author on reasonable request. The trial eligibility criteria annotation guideline is available in Additional file 1 .

## Abbreviations

API: Application Programming Interface
AUC: Area Under the Curve
BPA: Best Practice Advisory
CDM: Common Data Model
CDS-Hooks: Clinical Decision Support Hooks
CTSA: Clinical and Translational Science Awards
COVID-19: Coronavirus Disease 2019
DECOVRI: Data Extraction for COVID-19 Related Information
DW: Data warehouse
EHR: Electronic Health Record
FHIR: Fast Healthcare Interoperability Resources
GVHD: Graft versus Host Disease
MUSC: Medical University of South Carolina
n2c2: National NLP Clinical Challenges
NLP: Natural Language Processing
OHDSI: Observational Health Data Sciences and Informatics
OMOP: Observational Medical Outcomes Partnership
ROC: Receiver Operating Characteristic
TAES: TriAl Eligibility Surveillance
TREC: Text Retrieval Conference
UIMA: Unstructured Information Management Architecture
UMLS: Unified Medical Language System
UVB: Ultraviolet B
VT: Ventricular Tachycardia
WCTM: Watson Clinical Trial
