# Supplementary material for: Piloting an automated clinical trial eligibility surveillance and provider alert system based on artificial intelligence and standard data models
Source: BMC Med Res Methodol. 2023 Apr 11;23:88. doi: 10.1186/s12874-023-01916-6 (PMC10088225; doi:10.1186/s12874-023-01916-6)
Supplement: Supplementary file 1 — Additional file 1. [file 12874_2023_1916_MOESM1_ESM.pdf]

Trial Eligibility Annotation Schema

Translational Biomedical Informatics Center at MUSC

Last Updated: 2021-05-20 (v1.1)

Table of Contents

Introduction 2

- Conventions applied to classes and slots below 2
- Project Organization 2

Normalized Concept 3

- Condition or Disease 3
- Investigation Name 3
- Investigation Result/Value 4
- Medication Name 4
- Procedure 4

Demographics 5

- Age 5
- Gender 5
- Height 5
- Weight 5

Devices 6

Medication Allergy 6

Other Allergy 6

Attributes 6

- Conditional 6
- Generic 6
- Historical 6
- Negated 6
- Not Patient 7
- Uncertain 7

Change Log 7

Introduction

This document explains and provides guidelines for annotation of common inclusionary and exclusionary patient clinical trial eligibility criteria found in clinical notes. It also serves to maintain consistency across all annotators. The natural language processing tool will learn from the expert annotations and then automatically extract this information from electronic health records of various types. Annotations will be done to help localize spans of text that contain evidence for numerical scores and narrative evidence to support them. The annotation will be done using an online annotation tool: INCEpTION.

A PDF version of the rules, definitions, and examples in this project are available by clicking on the 'Guildelines' button at the top of any INCEpTION page.

Conventions applied to classes and slots below

In the provided examples, highlighted text provides examples of what to annotate. Beside the highlighted span is an information box showing the attribute(s) to set for the given span. Compare examples below. Good examples may appear in other formats not documented below but nontheless should be annotated. Implied evidence should not be included (e.g., '104.6F' implies a fever but does not explicitly state it).

Annotation	Text
Class 1	Filler text precedes the real annotation which can also be followed by fluff.
Class 2<br>– Attribute: Value	Some annotations are associated with attributes

Project Organization

In the provided examples, highlighted text provides examples of what to annotate. Click on the highlighted span to see how it was annotated (and to see any attributes associated with it). Good examples may appear in other formats not documented below but nontheless should be annotated. Implied evidence should not be included (e.g., '104.6F' implies a fever but does not explicitly state it).

The primary task for this project is to learn about the concepts definitions relevant for annotation. The layer containing all of these classes is called 'Entities'. There is a second layer called 'Relations' for drawing links between annotation (e.g., between a laboratory test name and its resultant value).

Some classes of information have drop-down menus for selecting a more specific type. Other classes of information require flipping a toggle box from 'No' to 'Yes'. We'll review each class of information in the rest of this project with examples already annotated. Below these examples is a 'Workshop' section to provide a space for you to try to match the annotation. If you find any examples ambiguous, please notify us and we can help clarify matters.

Annotation will be done in two phases. During the first phase, annotation is focused on finding instances of a concept type in the text. During the second phase, annotation is focused on normalizing instances in the text to an external standard concept. For instance, in Phase 1, your job would be to flag the span of text 'Prozac' as a **Medication Name** in the string 'Pt takes Prozac'. In Phase 2, your job would be to verify that the annotation for 'Prozac' was correctly mapped to the RxCUI 58827 in the RxNorm ontology. This document focuses on the concepts that you will be annotating in Phase 1.

Normalized Concept

Condition or Disease

Annotate the full mention of a disease, problem, or comorbidity. There may be **Negated** instances of comorbidities. Annotate them as **Negated** using the flag, as described in section on **Attributes**.

These concept will often be associated with the **Conditional** flag (when only present under certain circumstances like exercising), the **Historical** flag (when no longer present), **Negated** flag (when patient denies it), and the **Not Patient** flag (when a neighbor, spouse, friend, etc. is described as having it).

Annotation	Text
Normalized Concept<br>– Type: Condition or Disease	Pt has a history of <u>diabetes</u>…
Normalized Concept<br>– Type: Condition or Disease	and <u>sleep apnea</u>.

Investigation Name

Investigations and laboratory tests have two components to annotate: the name and the result or value. Annotate each component individually and then click-and-drag a relation arch between the two. The order (or direction) of the arrow does not matter. It is possible for an investigation to be mentioned without an appropriate result or value (e.g., when a lab is being ordered).

Body temperatures reported in the note should be annotated in this way unless it is described explicitly as a fever.

Annotation	Text
Normalized Concept<br>– Type: Investigation Name	reports a <u>temperature</u> of 103

(Nothing)	reports a fever of 103

Investigation Result/Value

Investigation results can be either categorical (e.g., 'positive', 'negative') or numerica (e.g., '120/80', '1.2 mg/dL'). In the latter case, include the units, when present, in the annotation. Remember to click-and-drag a **Relation** link between any given result annotation and the span of text mentioning the investigation name.

Annotation	Text
Normalized Concept<br>– Type: Investigation Result/Value	reports a temperature of 103

Medication Name

When a medication has multiple possible names (e.g., generic and brand names), annotate each one as a separate instance. This guideline is in contrast to a term and its acronym co-occuring together, which should be annotated as a single term (e.g., 'Gastroesophageal reflux disease (GERD)').

Some medication mentions are actually in terms of the medication being an allergen, as in the 'Levaquin' example below. Annotate these like other medications and then also slide the toggle box to a green 'Yes' beside **Medication Allergy** when annotating the name.

Medications that are no longer being taken should be flagged **Historical**, as described in the **Historical**. Medications that are being prescribed should be flagged **Uncertain**, as described in **Uncertain** section.

Annotation	Text
Normalized Concept<br>– Type: Medication Name	Acetaminophen (Tylenol or store brand)
Normalized Concept<br>– Type: Condition or Disease	Gastroesophageal reflux disease (GERD) occurs…

Procedure

As with **Conditions** and **Medications** (above), a **Procedure** may need to be further annotated with context flag like **Negated**, when a procedure was not performed.

Demographics

Age

Annotate any explicit mentions of age or date of birth.

Annotation	Text
Demographics – Type: Age	...a 35 year old (DOB: YYYY-MM-DD)
Demographics – Type: Age	...old (DOB: YYYY-MM-DD) male who…

Gender

Annotate any explicit mentions of gender (e.g., 'female' and 'male').

Annotation	Text
Demographics – Type: Gender	...a 35 year old (DOB: YYYY-MM-DD) male who…

Height

Annotate the numerical and unit component of any height mentions. That is, include the height and the units for the height.

Annotation	Text
Demographics – Type: Height	Height: 4 ft 6 in

Weight

Annotate the numerical and unit component of any weight mentions. That is, include the height and the units for the weight (usually 'lbs'). Indirect mentions (e.g., 'obese') should not be annotated.

Annotation	Text
Demographics – Type: Weight	Weight: 180 lbs

Devices

Annotate generic mentions of devices and brand names for common implanted medical devices. Due to the nature of the clinical trials we are focusing on, implanted devices need only be associated with one of three concept types:

- Left ventricular assist device
- Pacemaker
- Other device (for all other implanted devices)

Medication Allergy

Annotation	Text
Normalized Concept<br>– Type: Medication Name<br>– Medication Allergy: True	ALLERGIES: <u>Levaquin</u>

Other Allergy

Any other type of allergy mentioned for a patient (i.e., non-medication induced) should be indicated with this toggle box. The allergen may not even be annotated as one of the other types normalized concepts mentioned above (e.g., for seasonal allergies or pet allergies).

Attributes

Conditional

Problems that only occur under certain conditions (e.g., 'problems breathing while exercising') should be flagged as **Conditional**.

Generic

Flag concepts with **Generic** when they are mentioned in a generic way, not about any specific individual (e.g., Diabetes clinic).

Historical

Information from the patient's past and no longer present should be flagged as **Historical**.

Negated

Mentions with explicit negation should be flagged as **Negated** (e.g., 'patient denies abdominal pain')

Not Patient

Flag concepts with **Not Patient** when they apply to a specific individual who is not the patient (e.g., their spouse, roommate, or neighbor).

Uncertain

Annotated concepts should be flagged as **Uncertain** when they are possible or hypothetical mentions (e.g., 'I'm worried that…'), prescribed medications, or ordered (but not yet performed) investigations and procedures.

Annotation	Text
Medication<br>– Type: Name<br>– Uncertain: True	Azelastine 137 mcg (0.1 %) nasal aerosol… There are no refills with this prescription.

Change Log

- v1.1
  - Fixed a few typos
- v1.0
  - Added 2-stage annotation explanation
  - Fleshed out remaining concepts
- v0.1
  - Initial outline of concept types
